# Spectrophotometric Analysis and Optimization of 2D Gold Nanosheet Formation

**DOI:** 10.1021/acs.jpcc.2c07582

**Published:** 2023-02-06

**Authors:** Joseph Fox, George Newham, Richard J. Bushby, Elizabeth M. A. Valleley, Patricia Louise Coletta, Stephen D. Evans

**Affiliations:** † Molecular and Nanoscale Physics Group, School of Physics and Astronomy, University of Leeds, LeedsLS2 9JT, United Kingdom; ‡ School of Chemistry, University of Leeds, LeedsLS2 9JT, United Kingdom; § Leeds Institute of Medical Research, St James’s University Hospital, Wellcome Trust Brenner Building, LeedsLS9 7TF, United Kingdom

## Abstract

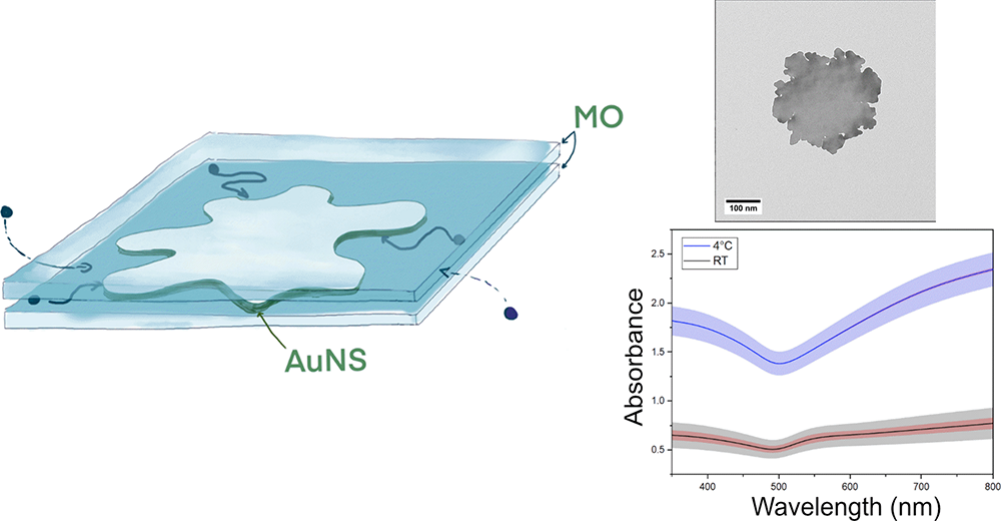

Free-standing, 2D gold nanosheets (AuNS) offer broad potential applications from computing to biosensing and healthcare. Such applications, however, require improved control of material growth. We recently reported the synthesis of AuNS only ∼0.47 nm (two atoms) thick, which exhibited very high catalytic activity. The synthesis is a one-pot, seedless procedure in which chloroauric acid is reduced by sodium citrate in the presence of methyl orange (MO). In this study, we use spectrophotometric analysis and TEM imaging to probe AuNS formation and optimize the procedure. Previously, we suggested that MO acted as the confining agent, directing two-dimensional growth of the gold. Here, we provide the first reported analysis of the HAuCl_4_ and MO reaction. We show that MO is rapidly oxidized to give 4-diazobenzenesulfonic acid, indicating that a complex interplay between HAuCl_4_, MO, and other reaction products leads to AuNS formation. Time-resolved studies indicate that synthesis time can be significantly reduced from over 12 to 2–3 h. Decreasing the reaction temperature from 20 to 4 °C improved AuNS yield by 16-fold, and the catalytic activity of the optimized material matches that obtained previously. Our elucidation of AuNS formation mechanisms has opened avenues to further improve and tune the synthesis, enhancing the potential applications of ultrathin AuNS.

## Introduction

Owing to their high physical and chemical stability, intrinsic biocompatibility, ease of surface functionalization, and unique optical properties, there has been a longstanding interest in gold nanoparticles (AuNP).^1^ As a result, AuNP have found extensive application in catalysis,^2^ drug delivery,^3^ sensing,^4^ medical imaging,^5^ and therapy.^6^ In several areas, including catalysis and sensing, the efficiency of the AuNP is related to the relative proportion of surface and edge atoms that are available for reaction.^7^ As such, using nanospheres or other 3D morphologies, where many gold atoms are occluded in the center of the AuNP, is undesirable and has led to increasing interest in developing two-dimensional (2D) gold nanostructures.

The synthesis of 2D gold nanostructures has proven non-trivial due to the natural tendency of metal atoms to form 3D close-packed lattice structures.^8^ Shin et al. obtained 2D branched gold nanostructures by adding hydroxylamine hydrochloride solution to HAuCl_4_, followed by slow addition of oleic acid.^9^ A color change was observed at the oleic acid/water interface, and material below the interface was collected. This method obtained 2D particles with nanodendrimer and nanourchin morphologies (time-dependent) with thicknesses of ∼5 nm.^9^ Niu et al.^10^ obtained gold nanosheets of thickness 3.6 nm by using a novel lamellar hydrogel as a soft 2D template. Marangoni et al. produced 2D gold nanosheets by reducing gold seeds adsorbed onto bipyridine functionalized graphene oxide nanosheets. Altering the seed concentration allowed tunable synthesis of ultra-thin gold nanosheets (∼5 nm thick) without using stabilizers or surfactants.^11^ Seedless generation of 2D gold nanostructures has been achieved by Momeni et al.^12^ using red marine alga as a shape-directing agent to generate gold nanosheets of thickness 10–15 nm. HAuCl_4_ aqueous solution was mixed with crude extract of red alga and stirred for 10 h at room temperature before product collection. An analogous synthesis method was employed by Shankar et al.,^13^ using lemongrass extract combined with a gold precursor to generate gold nanotriangles (∼25 nm thick). In both studies, pre-processing of the shape-directing agent is required and the mechanism through which the shape-directing agent acts is not elucidated.

**Figure 1:**
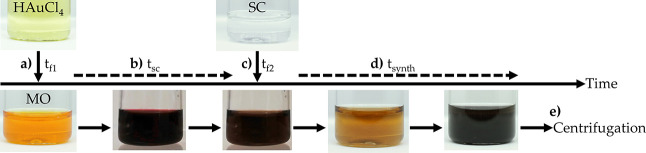
Schematic of the steps involved in the synthesis of 2D AuNS. (a) HAuCl_4_ addition, feed time *t*_f1_. (b) Reaction time *t*_sc_, before the addition of SC. (c) SC addition, feed time *t*_f2_. (d) Reaction proceeds at room temperature for time *t*_synth_. (e) Products collected by centrifugation.

We recently established a protocol for the seedless synthesis of sub-nanometer (0.47 nm-thick, two-atom-thick) 2D gold nanosheets (AuNS).^8^ In this one-pot synthesis, chloroauric acid (HAuCl_4_) was reduced by trisodium citrate (SC) under ambient conditions in the presence of methyl orange (MO), as outlined in Figure 1. MO is a highly polar amphiphile with a rigid hydrophobic core. At low concentrations, it can self-assemble in water to form chromonic liquid crystalline phases.^8,14^ Our original understanding was that it was this self-assembly behavior of MO, even at very low concentrations, that was responsible for promoting the 2D growth of the AuNS. When MO was omitted from the synthesis, 3D AuNP were formed. However, the role of MO is more complex than acting as a simple “confinement” agent, and the aim of this study was to look more deeply into the mechanisms associated with the formation of 2D AuNS using a range of spectroscopic methods coupled with TEM imaging. In particular, the reagent feed times (*t*_f1_ and *t*_f2_), the time delay before the addition of the SC (*t*_sc_), and the overall reaction time (*t*_synth_) were explored. These parameters, shown in Figure 1, together with temperature and subsequent cleaning schemes, are critical in controlling the yield and quality of the final AuNS product. Our analysis of the synthesis has resulted in a more robust, reproducible protocol delivering maximized AuNS yield and additional insights into AuNS formation mechanisms.

## Methods

### Materials

Gold(III) chloride trihydrate (520918) and potassium bromide (KBr, 221,864) were purchased from Sigma. MO (17874) and trisodium citrate (45556) were purchased from Alfa Aesar. Hydrochloric acid (32%, H/1100/PB17), nitric acid (70%, N/2250/PB17), sodium borohydride (S/2560/46), and 20 mL borosilicate clear glass vials (14-955-313) were purchased from Fisher Scientific. 4-Nitrophenol (73560) was purchased from Fluka Analytical. Milli-Q water (18.2 MΩ.cm at 25 °C) was used in all solutions/synthesis.

### General AuNS Synthesis Protocol

To synthesize AuNS, 4 mL of 0.21 mM MO was added to an aqua regia-cleaned glass vial at room temperature, followed by 1 mL of 5 mM HAuCl_4_ (*t*_f1_ = 1 s). After 30 s (*t*_sc_), 500 μL of 100 mM sodium citrate was added (*t*_f2_ = 1 s). The synthesis mixture was left undisturbed for 12 h at room temperature before being washed three times by centrifugation (1000 *g* for 10 min) and resuspending the pellet in 2 mL of Milli-Q water then 1 mL for the two subsequent rinsing steps. To study and optimize the synthesis protocol, the time interval (*t*_sc_), the reagent feed times (*t*_f1_ and *t*_f2_), the reaction time (*t*_synth_), the synthesis temperature (*T*), and the centrifugation speed were systematically varied without alteration to the reagent concentrations or order of addition.

### AuNS Characterization

Absorbance spectra were taken between 200 and 800 nm using an Agilent Technologies Cary 5000 UV–vis–NIR spectrophotometer. TEM imaging was conducted using two systems: (i) a Tecnai G2 Spirit TEM (T12), operated at an acceleration voltage of 120 kV with a Lab6 filament and a Gatan Us4000 CCD camera for image capture, and (ii) an FEI Tecnai TF20 FEGTEM operated at an acceleration voltage of 200 kV with a Gatan Orius SC600A CCD camera for image capture. Grids used for imaging were 400 mesh copper grids coated with a ∼8 nm-thick carbon support film (SPI Supplies). Samples were prepared by pre-treating carbon-coated copper grids with PELCO easiGlow, followed by adding 3 μL of nanoparticle solution (in Milli-Q) pipetted onto the grid and left to dry. In cases where the absorbance of a cleaned sample was above an optical density at 400 nm (OD_400_) OD_400_ = 1, samples were diluted in Milli-Q to OD_400_ = 1 before TEM imaging. Fourier transform infrared (FTIR) spectroscopy analysis was performed using the Bruker IFS 66v/s system using a deuterated triglycine sulfate (DTGS) detector with aperture and scanner velocity of 6 mm and 10 kHz, respectively. Five hundred scans per sample were taken over a wavenumber range of 4000–400 cm^–1^, with 2 cm^–1^ resolution. KBr discs were made using a manual hydraulic press (Specac Ltd.) to apply 9000 kg of pressure, to 200 mg of ground KBr, for 3 min under a constant vacuum. Blank KBr discs were used for background measurements, while sample discs were made by mixing solid sample material with 200 mg of ground KBr before the pressing process. Background spectra have been removed from all spectra, and baseline corrections have been performed.

### UV–Vis Analysis of the MO and HAuCl_4_ Reaction

A Hellma Analytics (104-650-10-41) 4 mm pathlength quartz cuvette was cleaned with aqua regia for 30 min, rinsed thoroughly with Milli-Q, and dried with nitrogen gas. The reaction volumes were scaled down fivefold to follow the synthesis spectroscopically. Spectral regions of interest, 240–320 and 420–540 nm, were scanned every ∼8 s over a 2 min period. Fast-scan analysis was conducted by placing a cuvette containing 767 μL Milli-Q with 33 μL of 5 mM MO (pipette mixed) into the instrument, quickly injecting 200 μL of 5 mM HAuCl_4_ and beginning the scanning kinetics sequence. All reagents were used at room temperature. Fast-scan data sets for regions 240–320 and 420–540 nm were obtained in triplicate, with cuvettes aqua regia cleaned between sets, to remove residual gold.

### FTIR Analysis of the MO and HAuCl_4_ Reaction

For FTIR analysis of the reaction between MO and HAuCl_4_, the reaction product was freeze-dried to obtain a solid sample for mixing with KBr. Briefly, 4 mL of 0.21 mM MO was mixed with 1 mL of 5 mM HAuCl_4_ (*t*_f1_ = 1 s) and allowed to react at room temperature for 30 s. Three samples were “flash” frozen in liquid nitrogen for 2 min and then placed into a Mini Lyotrap bench top freeze dryer (LTE Scientific, Ltd.), pre-cooled to −55 °C, and kept under 9 × 10^–3^ mbar vacuum overnight using a Scrollvac 10 plus pump (Leybold).

### Catalytic Assessment of AuNS

To assess the catalytic performance of the AuNS, 10 μL of 15 mM 4-nitrophenol, 980 μL of 20 mM sodium borohydride, and 10 μL of AuNS (300 μg mL^–1^) were added to a cuvette and mixed. The reaction was monitored using optical absorbance at 400 nm over time. The apparent rate constant, *k*_app_, was calculated using pseudo first-order kinetics and is given using eq 1 and the mass normalized rate constant, *k*_1_, using eq 2:
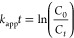
1
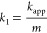
2

*C*_0_ and *C_t_* are the concentrations of 4-nitrophenol, determined from the 400 nm absorbance at time 0 and *t*, respectively, and *m* is the AuNS mass. The experiment was conducted in triplicate.

## Results and Discussion

### Interaction of HAuCl_4_ with Dilute MO Solution

Our original understanding was that MO behaved as a confining agent, directing the formation of the ultra-thin gold. No MO was observed in the collected product, and the synthesis of 2D AuNS was critically dependent on the concentration of MO used.^8^ However, the process of AuNS formation seems to be more complex, with the final product being dependent not only on concentrations of the reactants but also on the time and speed of reagent addition. The addition of HAuCl_4_ to MO leads to a rapid color change (Figure 1), initially to a dark red color then to nearly black, which subsequently lightens over time. The time for citrate addition was previously determined by eye based on the color of the solution. To better understand and control this aspect of the formation process, we have analyzed this interaction with UV–vis spectroscopy.

**Figure 2:**
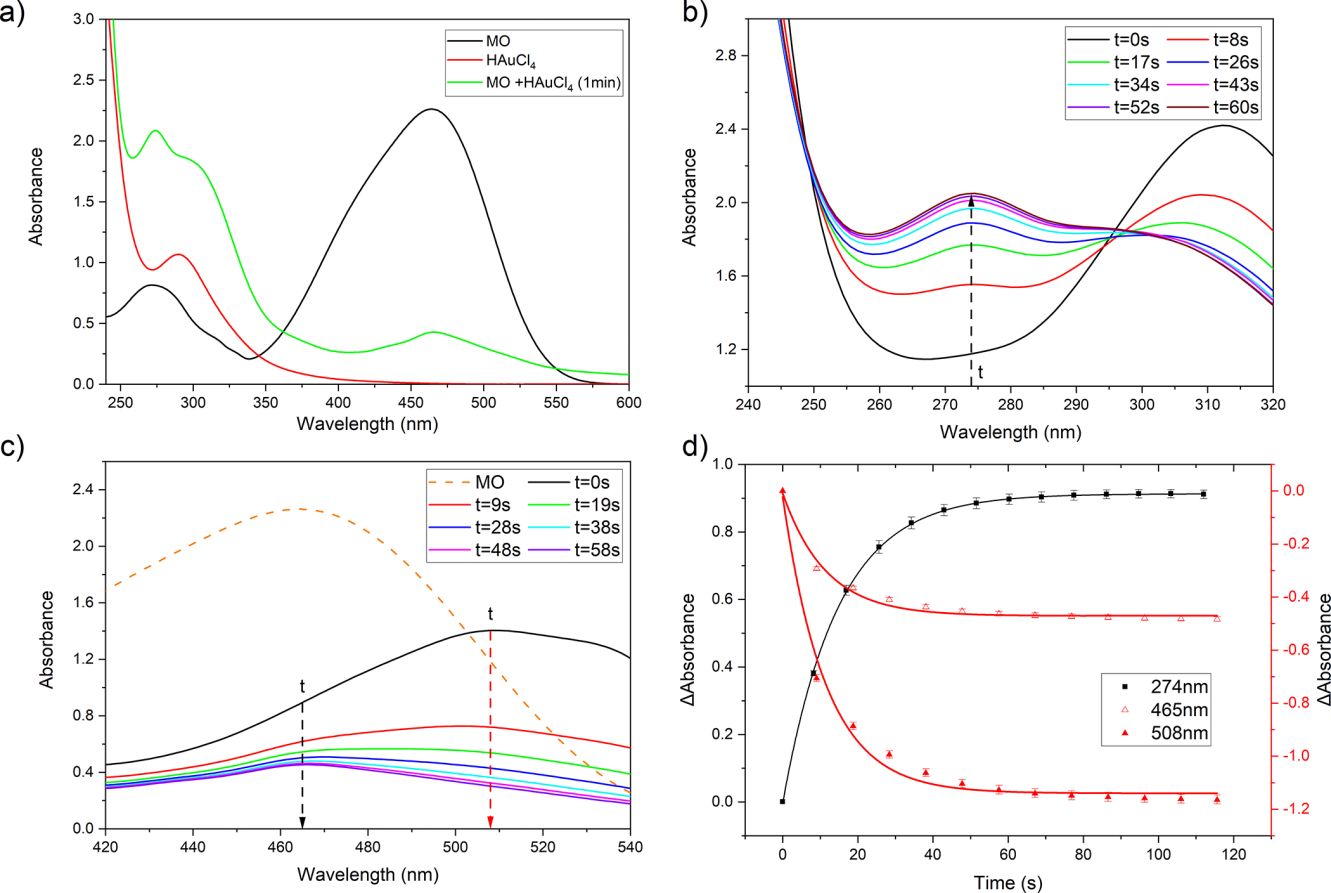
Analysis of the reaction between methyl orange and HAuCl_4_. (a) UV–vis spectra of MO and HAuCl_4_ prior to mixing and MO 1 min after the addition of HAuCl_4_. (b) and (c) show time-resolved UV–vis spectra of the MO + HAuCl_4_ reaction in regions 240–320 and 420–540 nm, respectively, at given time points, with *t* = 0 s representing the instant MO and HAuCl_4_ are mixed and wavelengths of interest (274, 465, and 508 nm) denoted with dashed arrows. (d) Change in absorbance (ΔAbsorbance) with time at 274, 465, and 508 nm. ΔAbsorbance is calculated with respect to absorbance at first time point of reaction, averaged over three repeats and exponential decay fit applied.

Figure 2a shows UV–vis spectra of HAuCl_4_, MO, and MO + HAuCl_4_, after 1 min of reaction, at the same concentrations used for AuNS preparation. After 1 min, the spectrum of the mixture shows a dramatic decrease in the MO band at ca. 450 nm, an absorbance at 290 nm due to HAuCl_4_, and a new peak at 274 nm.

**Scheme 1:**
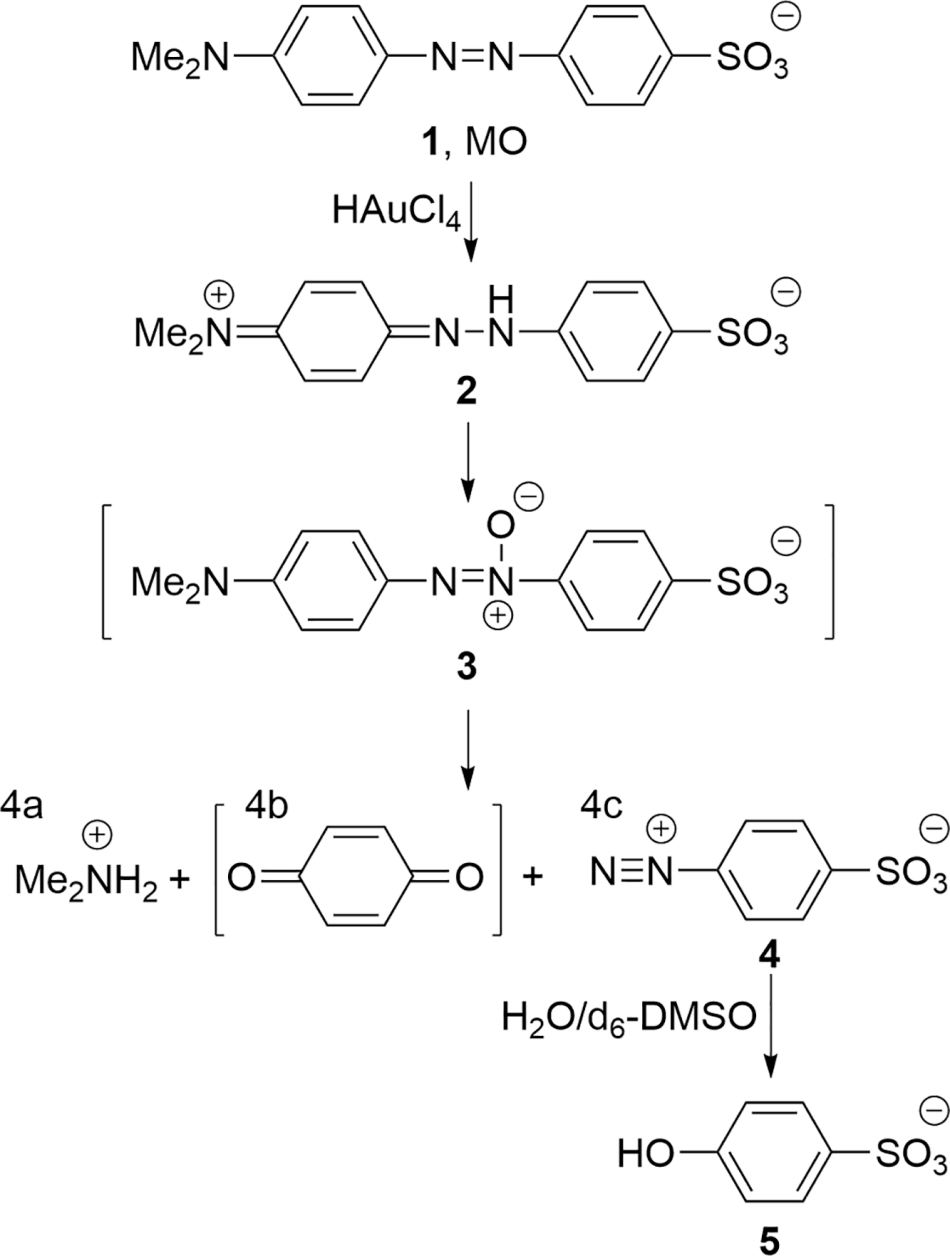
Proposed Reaction Scheme for the Interaction of Methyl Orange with HAuCl_4_

To analyze the process in more detail, the spectral regions 240–320 and 420–540 nm were selected for fast-scan analysis (Figure 2b and c, respectively). The spectra in Figure 2b show a broad shoulder at 310 nm diminishing as a new peak at 274 nm appears. Over the same period, we observe that the strong absorbance band at 465 nm associated with MO alone (orange dashed line, Figure 2c) undergoes an almost instantaneous shift to a peak at 508 nm (black solid line, Figure 2c) upon the addition of HAuCl_4_, associated with the protonation of the N=N bond in MO, leading to compound **2** (Scheme 1) ( Figure 2c, *t* = 0 s). This shift due to protonation of MO is in agreement with published studies^15^ and can be seen in our spectra for the addition of HCl to MO, shown in Figure S1a. The protonation occurring when MO and HAuCl_4_ interact is followed by a rapid, pseudo first-order reaction, which is almost complete after 30 s, in which a new band appears at 465 nm, and the band at 508 nm associated with the protonated MO is progressively reduced. Figure 2d shows the change in absorbance for the peaks at 274, 465, and 508 nm as a function of time. The changes in absorbance plateau after ∼30 s, indicating that the reaction between MO and HAuCl_4_ is essentially complete in 30 s. Exponential fits to these plots establish that the process is pseudo first-order (which is expected since HAuCl_4_ is present in excess), and all yield similar time constants, τ = 11.5 ± 0.9 s, corresponding to the same reaction. We believe these spectral changes reflect the degradation of the MO and are consistent with literature studies.^16−21^ Images of the time-dependent color change are shown summarily in Figure 1 and in more detail in Figure S1b.

To establish the nature of the products formed, proton nuclear magnetic resonance (^1^H-NMR) was performed on a sample of reacted MO/HAuCl_4_. The ^1^H-NMR spectrum of the total product dissolved in D_2_O showed doublets at 8.74 and 8.31 δ (*J*, coupling constant = 9.0 Hz) due to the aryl hydrogens of the diazonium compound and a singlet at 2.73 δ assigned to the *N*-deuterated dimethylammonium ion. The spectrum in d_6_-DMSO showed doublets at 8.65 and 8.11 δ (*J* = 8.8 Hz) due to the aryl hydrogens of the diazonium compound, and a triplet at 2.55 δ (*J* = 5.6 Hz) is assigned to Me_2_NH_2_^+^. The spectra in d_6_-DMSO were compared to those of 4-DBSA, prepared by nitrous acid treatment of sulfonamide,^22^ and these are shown in Figure S2a–c. A comparison of the two spectra shows several (unidentified) minor products in addition to the major oxidation products, protonated dimethylamine and 4-diazobenzenesulfonic acid (4-DBSA), compounds **4a** and **4c**, respectively (Scheme 1).

**Figure 3:**
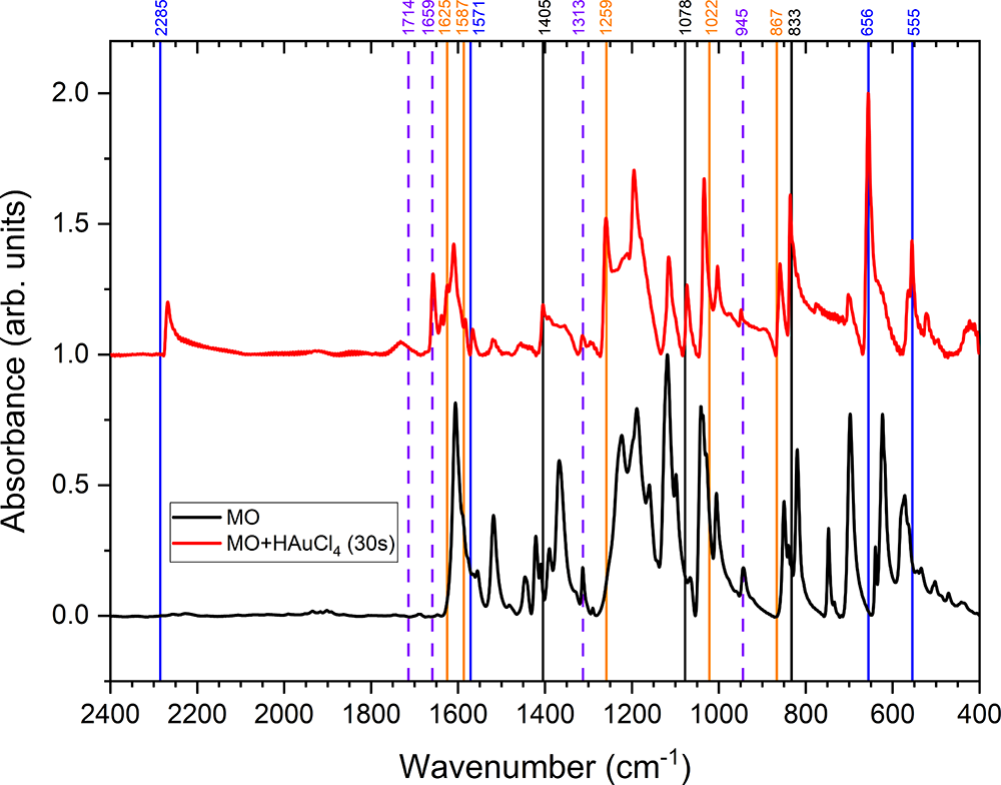
FTIR analysis of the interaction of HAuCl_4_ with MO. The colored vertical lines correspond to expected reaction products 4-DBSA (dark blue), dimethylammonium ion (orange), and *para*-benzoquinone (violet, dashed). Black vertical lines indicate peaks expected in multiple reaction products.

Additional experimental support for Scheme 1 comes from FTIR spectroscopy. Figure 3 shows the mid-frequency region for MO and MO/HAuCl_4_ (after 30 s of reaction). The vertical lines indicate peak positions for the main products as obtained from the literature for dimethylammonium ion (**4a**, Scheme 1) and *para*-benzoquinone (**4b**, Scheme 1)^24,25^ and our own spectra (Figure S3) for 4-DBSA (**4c**, Scheme 1). The black vertical lines at 833 and 1405 cm^–1^ can be assigned to dimethylammonium ion and 4-DBSA, while the black vertical line at 1078 cm^–1^ can be assigned to all three reaction products. The appearance of the new peak ca. 2267 cm^–1^ is ascribed to the presence of the N≡N triple bond^23^ and is consistent with the production of 4-DBSA.

The UV–vis spectrum for 4-DBSA (Figure S1a, scaled to a concentration of 0.21 mM) has a peak at 269 nm of similar intensity to that seen following the reaction of the MO and HAuCl_4_ (1 min) spectra. This also agrees with literature values for the absorbance of 4-DBSA.^24^ The combination of NMR, FTIR, and UV–vis data would support the idea that the interaction of MO with HAuCl_4_ leads to degradation of the MO with the diazonium compound 4-DBSA (**4c**, Scheme 1) likely as the main oxidation product. The formation of a diazonium salt by oxidation of an azo dye is unusual chemistry, but it is not unprecedented.^25^ Most cases where this sort of chemistry is observed involve the oxidation of an acid solution of an azo dye in which one of the Ar-N=N-Ar aryl residues is very electron-rich (with an *ortho* or *meta* MeO, HO, or Me_2_N substituent) and the other aryl residue is very electron poor (with a strongly electron-withdrawing substituent^25^). It is assumed that the first step is the formation of the azoxy compound Ar-N=N(O)-Ar where an oxygen has been added to the nitrogen next to the electron-poor ring—compound **3** in the case of MO (Scheme 1).

Overall, these studies show that, under the conditions used to prepare the ultra-thin gold, HAuCl_4_ is a sufficiently strong oxidant to rapidly oxidize the MO to 4-DBSA (**4c**, Scheme 1) and that it is this, rather than the MO, that may be the critical “confining” agent; this might be expected, since diazonium salts are used to modify metal surfaces and electrodes.^26,27^

To test our hypothesis, we tried reactions in which a 0.21 mM solution of 4-DBSA (synthesized from sulfonamide^22^) was used rather than a 0.21 mM solution of MO. This synthesis yielded structures with characteristic wrinkling of the sheet surface (a hallmark of ultrathin 2D material); however, a significant quantity of 3D material was also present, and no standalone AuNS (Figure S4a-d). This result indicates the importance of the self-assembly of the MO before its in situ oxidation on interaction with HAuCl_4_. Hence, in the following experiments, we analyzed preparations of AuNS in which the citrate was added either immediately after HAuCl_4_ (*t*_sc_ = 0 s) or after sufficient time for all the MO to be reacted (*t*_sc_ = 30 s).

MO is thought to have a dual role in AuNS formation. First, due to its well-known chromonic-phase liquid crystal behavior,^14^ it is known to undergo planar stacking, at extremely low concentrations, in aqueous solutions. We believe that this stacking acts as a template for controlling the 2D nature of the growth, similar to that reported by Niu et al.^10^ using lamellar-based hydrogels. However, there is a second proposed aspect of the role of MO, in which MO restricts the growth of the gold in the [111] direction by preferentially adsorbing to these facets.^8^

We propose that the Au^3+^ ions interact with the aromatic rings of the MO^28,29^ and are subsequently reduced, while the MO undergoes concomitant oxidation and is broken down into a number of reaction products. The reduced Au-oxidized MO complex serves as a nucleus for metallic Au growth, driven by the addition of SC, while also stopping growth in the [111] direction. This might be similar in mechanism to the role played by iodide ions, or agents used in other 2D Au synthesis.^30^ The fractal-like appearance of the 2D sheets is consistent with the addition of new gold at sheet edges being controlled via a diffusion-limited aggregation pathway.^31^

### Role of MO/HAuCl_4_ Interaction Time before Sodium Citrate Addition (*t*_sc_)

To investigate the effect of the MO/HAuCl_4_ interaction reaching completion before the addition of the SC, we took spectra as a function of time for two conditions: (a) simultaneous addition of HAuCl_4_ and SC into the MO, *t*_sc_ = 0 s, and (b) a gap of 30 s between the MO/HAuCl_4_ mixing and the addition of the SC, *t*_sc_ = 30 s.

**Figure 4:**
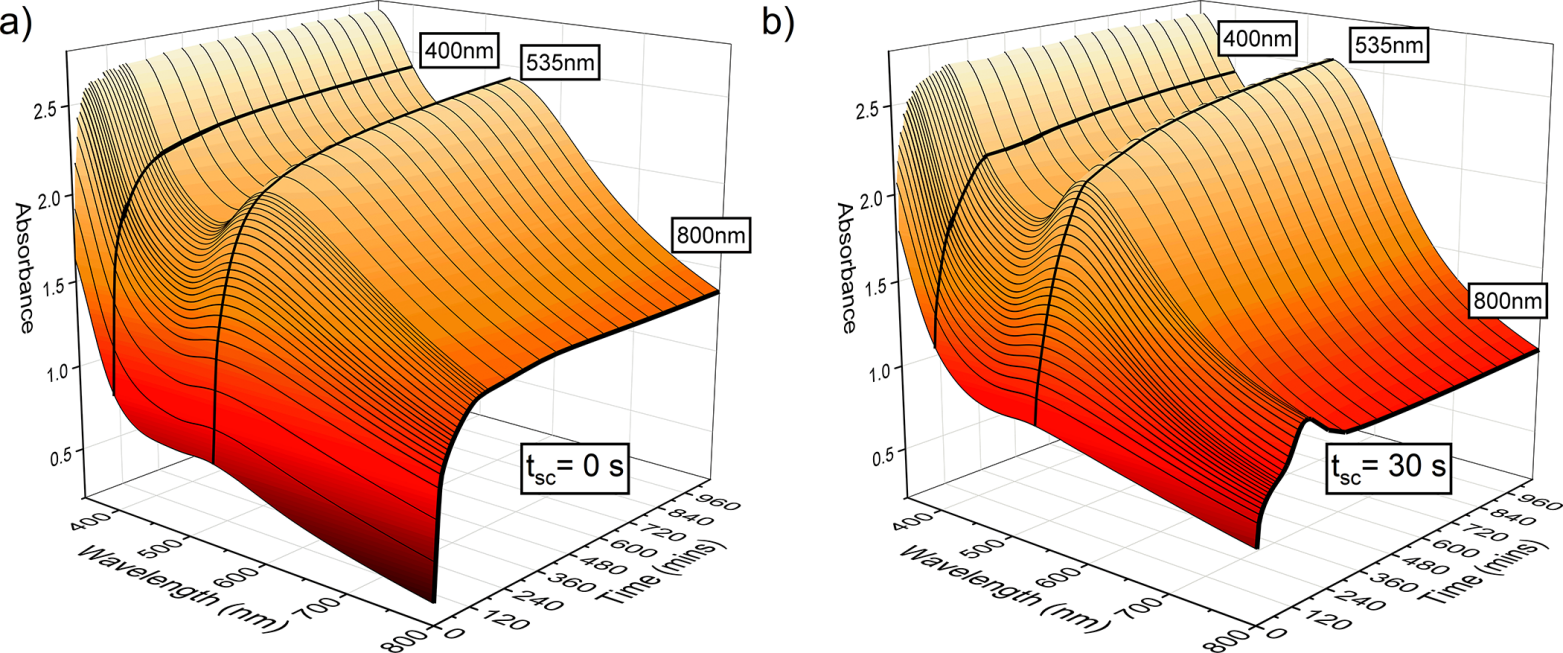
Time-resolved UV–vis analysis of synthesis (*t*_sc_ = 0 or 30 s, *t*_f1_ = *t*_f2_ = 1 s, and *t*_synth_ = 18 h). Time-resolved UV–vis surface plots for synthesis monitored over a reaction time of 18 h, in which (a) *t*_sc_ = 0 s and (b) *t*_sc_ = 30 s. In both plots, bold lines indicate wavelengths of interest 400, 535, and 800 nm.

Figure 4a,b shows spectral evolution over time for the two conditions. Three key wavelengths are indicated in bold on the figure, at 800, 535, and 400 nm, and are associated with the 2D, 3D, and total reduced (metallic) gold, respectively. In both cases, the plasmon resonance at 535 nm, indicative of 3D AuNP formation, increases monotonically with time. The absorbance at 800 nm, taken as an indicator of the presence of 2D AuNS, increases and plateaus, after ∼2 h, in the case of simultaneous addition (*t*_sc_ = 0 s) of the reagents, while in the *t*_sc_ = 30 s case, the 800 nm band reaches a peak after ∼3 h and proceeds to decrease before plateauing. This implies that different formation mechanisms and reaction rates are at play in the *t*_sc_ = 0 and 30 s cases. Considering only the *t*_sc_ = 30 s regime, the decline in the 800 nm band indicates a loss in the amount of 2D gold from the solution. Since no precipitation is observed, it would appear that some of the 2D AuNS is either not stable against breakdown into Au ions or, perhaps more probably, transitioning to 3D growth. The absorbance at 800 nm was consistently higher, at all time points, for *t*_sc_ = 0 s compared to *t*_sc_ = 30 s, indicative of more 2D gold being produced. The spectra do not alter significantly after 4 h, suggesting the potential to reduce the time required to form AuNS material significantly. Hence, ending the synthesis before this point may permit collection of more 2D gold and improve structural purity. It is evident from Figure 4 that allowing the formation of 4-DBSA (i.e., the *t*_sc_ = 30 s condition) before the addition of sodium citrate increases the relative concentration of 3D material in comparison to 2D material, as indicated by the ratio of absorbance at 535 and 800 nm, respectively.

Centrifugation was used to separate 3D AuNP from 2D AuNS. The effect of centrifugation on both the *t*_sc_ = 0 and 30 s formation conditions is presented in Figure S5. The results show that in the *t*_sc_ = 0 s case, increasing spin speed gave a higher yield of total gold product (OD_400_); however, in all cases, spectral shape and particle morphology (mixture of a 2D material and associated 3D-structured material, see Figure S5a) did not change significantly. In contrast, for the *t*_sc_ = 30 s case, the spin speed was shown to alter spectral and morphological characteristics. For *t*_sc_ = 30 s, stronger spin speeds produced spectra with lower absorbance at 800 nm (linked to 2D AuNS) and a more pronounced surface plasmon resonance (SPR) band ∼535 nm (linked to 3D structures); see Figure S5b. In the 4600 *g* case, we observe tape-like particles, which in some cases display a dense 3D head (Figure S5e), explaining the emergence of the SPR band in the particle spectra. At lower spin speeds (1000 *g*), particle spectra showed improved NIR absorbance and yielded larger structures with 2D regions (Figure S5c). Hence, the desired AuNS morphology was obtained using *t*_sc_ = 30 s with centrifugation at 1000 *g*; these parameters will be used for further study.

High-resolution TEM (HRTEM) and atomic force microscopy (AFM) measurements of AuNS were reported in our previous study.^8^ HRTEM of AuNS showed a sixfold symmetric structure with lattice spacing of 0.25 nm. HRTEM and selected-area electron diffraction (SAED) suggest the single-crystalline structure of AuNS with a < 111> orientation. AFM studies revealed AuNS to be atomically flat, with a thickness of 0.47 nm. Taken together, HRTEM and AFM suggest that AuNS consists of two atomic layers of Au atoms. The apparent variation in thickness and morphology visible in TEM images is likely caused by clustering and folding of AuNS during the drying of 2D gold onto TEM grids. Some control over the lateral dimension is possible by controlling the reaction time and is the subject of ongoing studies. Storage of AuNS structures in solution, at room temperature in the dark, yields stability for over 15 months.^8^

### Influence of Reagent Feed Time (*t*_f1_ and *t*_f2_) on AuNS Formation

It was previously suggested that the role of MO was as a structure-directing agent due to the formation of planar MO stacks that template the 2D AuNS formation.^8^ The fast injection of reagents and concomitant turbulent mixing might disrupt this liquid crystalline order, reducing the yield of AuNS. Hence, we evaluated the effect of reducing the reagent feed times of the HAuCl_4_ and SC solutions. To reduce the parameter space explored, *t*_f1_ was kept equal to *t*_f2_ and are referred to by *t*_f_.

**Figure 5:**
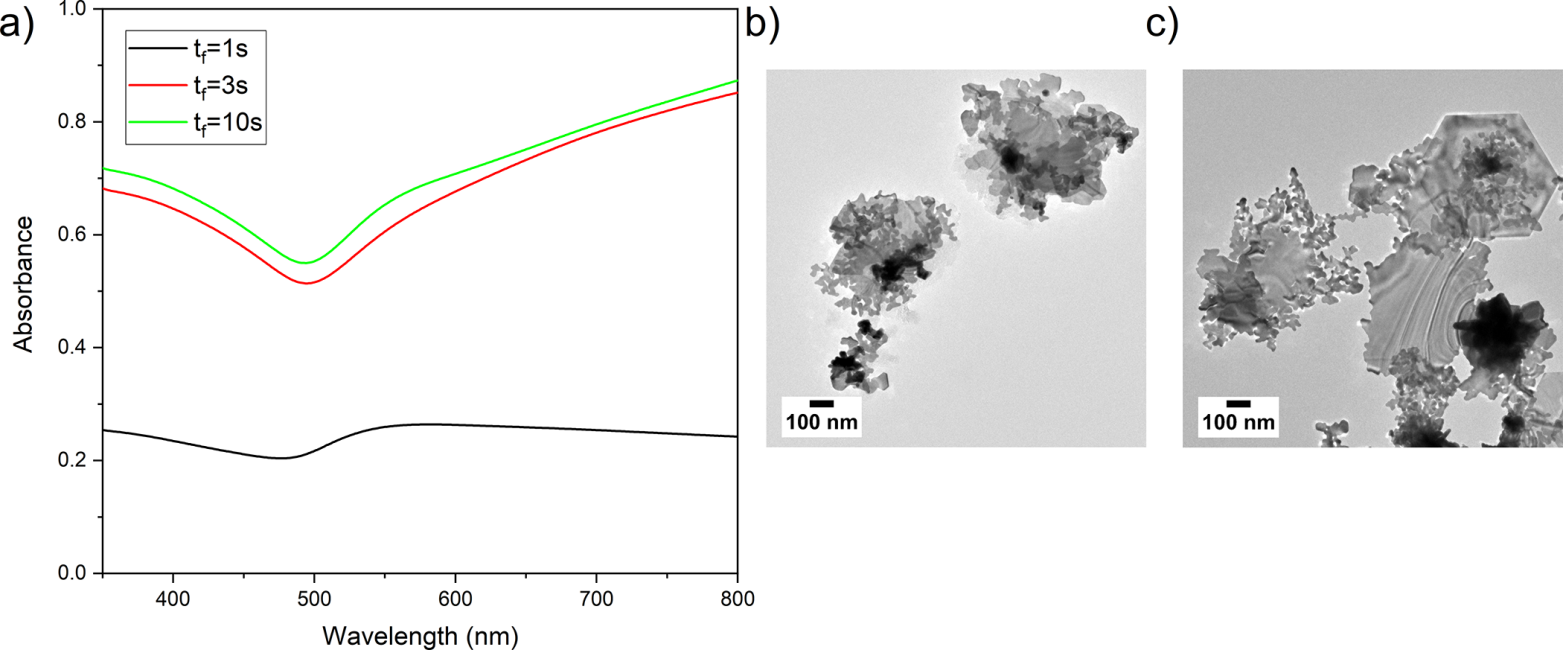
Influence of reagent feed time (*t*_f_) (*t*_sc_ = 30 s, *t*_synth_ = 16 h 30 min, centrifugation 3× at 1000 *g* for 10 min). (a) Post-clean UV–vis spectra of samples synthesized using different reagent feed times. F20 TEM images of post-clean samples, which received (b) *t*_f_ = 3 s and (c) *t*_f_ = 10 s.

Figure 5a shows the post-clean UV–vis spectra of AuNS as the rate of HAuCl_4_ and SC addition was varied. Slow addition of the reagents (pipetted over 3 or 10 s) led to a near fourfold increase in 2D AuNS yield compared to a fast injection (pipetted over 1 s). Furthermore, slower feed rates led to the formation of larger AuNS, as shown by TEM (Figure 5b,c) with Figure 5a showing corresponding TEM for fast injection. This implies that by reducing turbulent mixing, larger structures form, which pellet out with the relatively gentle 1000 *g* spin. When the reagents were added more slowly, two distinct layers were observed with a dark meniscus forming on the surface of the liquid. After ∼10 min, this layer sank to the bottom. The spectra and morphology of *t*_f_ = 3 and 10 s samples was similar, indicating that it is not the feed time that is key but the lack of a vigorous mixing effect upon reagent addition; hence, *t*_f_ = 3 s was carried into future experiments.

### Effect of Total Synthesis Time (*t*_synth_) on AuNS Formation

**Figure 6:**
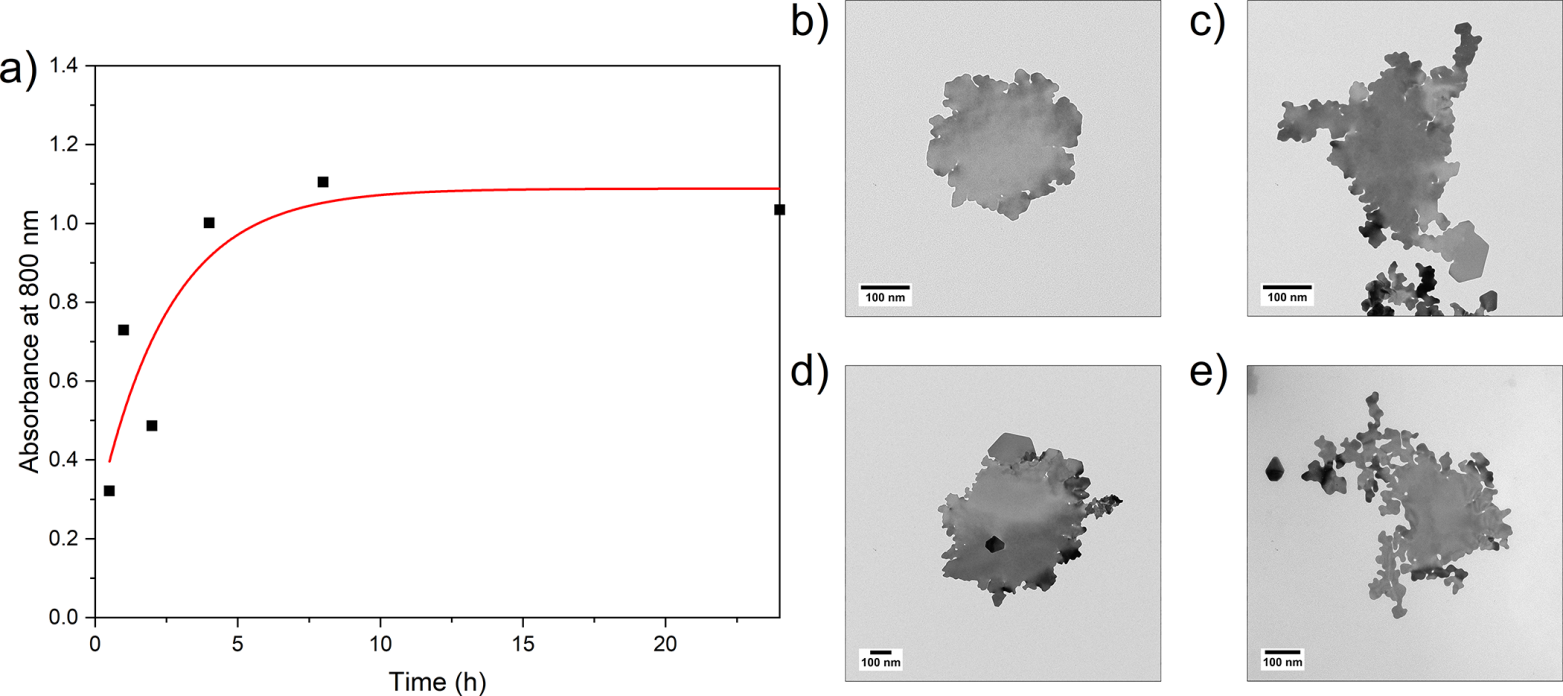
Influence of *t*_synth_ (*t*_sc_ = 30 s, *t*_f_ = 3 s, centrifugation 3× at 1000 *g* for 10 min). (a) Post-clean absorbance at 800 nm with respect to time. T12 TEM images of the post-clean product when *t*_synth_ was (b) 1 h, (c) 2 h, (d) 4 h, and (e) 24 h.

We had previously suggested that the reaction be allowed to proceed for >12 h to obtain optimal yield of 2D AuNS. Here, we show how spectra, yield, and morphology alter with synthesis time (*t*_synth_). Spectra were obtained for samples produced with *t*_f_ = 3 s and *t*_sc_ = 30 s for reaction times between 0.5 and 24 h. The pre-clean spectra shown in Figure S6a show the characteristic SPR peak for 3D AuNP at ∼535 nm becoming more pronounced with time while the 2D AuNS plateaus after ∼200 min. This can indicate that ending the reaction sooner may improve the structural purity of the collected 2D AuNS. Post-clean UV–vis spectra are shown in Figure S6b. The spectra are sensitive to centrifugation/collection conditions but show a general trend of increasing 2D content with synthesis time, as represented in Figure 6a by the change in absorbance at 800 nm with time. We observe that the 2D AuNS yield plateaus at ∼8 h. TEM taken at different endpoints (Figure 6b–e) shows no significant morphological changes over this period, with AuNS structures observed after 1 h, but with low yield (OD_400_).

### Influence of Synthesis Temperature (*T*) on AuNS Formation

**Figure 7:**
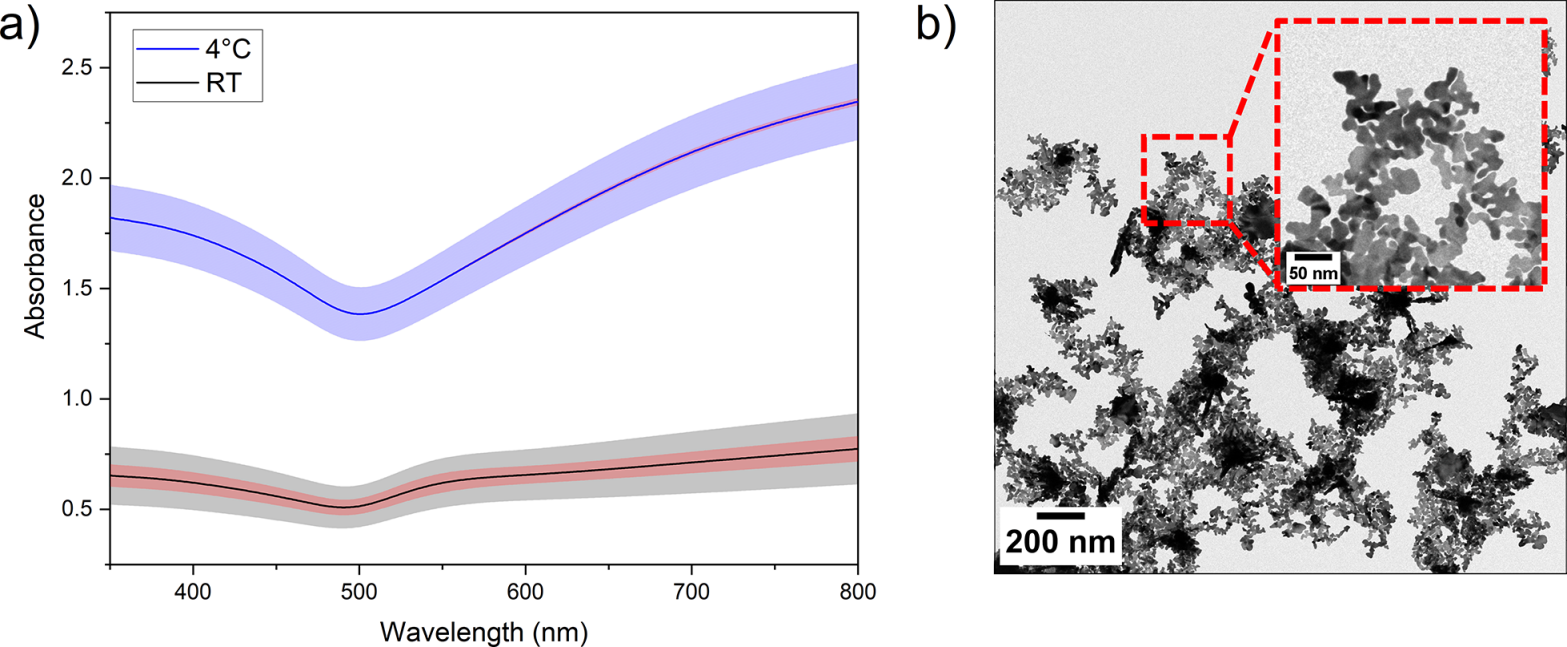
Influence of synthesis temperature (*t*_sc_ = 30 s, *t*_synth_ = 16 h, centrifugation 3× at 1000 *g* for 10 min). (a) Post-clean UV–vis spectra showing average of three repeat syntheses at either room temperature with *t*_f_ = 3 s and *t*_sc_ = 30 s or 4 °C with *t*_f_ = 1 s and *t*_sc_ = 30 s, with standard deviation shown by color-matched shaded regions. Red-shaded regions show standard deviation of average spectra post-normalization. (b) TEM image of AuNS formed at 4 °C with *t*_f_ = 1 s and *t*_sc_ = 30 s.

Here, we compared the addition of reagents at room temperature (RT) and 4 °C. In Figure 5a, it was shown that when *T* = RT, *t*_f_ = 3 s offered an improved yield over *t*_f_ = 1 s. However, it was also established that when *T* = 4 °C, *t*_f_ = 1 s provided increased yield over *t*_f_ = 3 s. As the different addition speeds gave different yields at different temperatures, we compared the optimal addition speed for each temperature. Figure 7a shows the average from triplicate syntheses conducted at room temperature (*t*_f_ = 3 s) and at 4 °C (*t*_f_ = 1 s). The triplicate spectra have been averaged, and the blue- and black-shaded regions show standard deviation. The reduced temperature synthesis led to a near threefold increase in OD_400_ (used to represent total gold yield) while maintaining the characteristic UV–vis spectra for 2D gold. It is likely that the reduced temperature favored the slow growth of 2D sheets over the rapid formation of new nuclei and 3D material associated with high reaction rates. This agrees with Baral et al., who observed that a reduction in reaction rate led to the formation of larger 2D nanostructures.^32^ The conditions (*t*_sc_ = 30 s, *t*_f_ = 1 s, *T* = RT, 1000 *g* clean) from our previous AuNS publication^8^ yielded OD_400_ = 0.11 (Figure S5b) after cleaning. In comparison, our optimized protocol, conducted at 4 °C, gives OD_400_ = 1.74 (Figure 7a) after cleaning, representing an ∼16× increase in gold mass. To represent variation in spectral characteristic, the red-shaded region shown in Figure 7a shows standard deviation of the triplicate spectra calculated after normalization to OD_400_. Hence, we observe that reduction in synthesis temperature decreased spectral variation. Morphological analysis by TEM showed that under the reduced temperature condition, high concentrations of 2D AuNS were observed (Figure 7b) and the absence of the SPR peak at ∼535 nm (Figure 7a) suggests that any 3D structures observed by TEM are aggregates of the 2D material. Consistent with our previous work,^8^ the edges of the AuNS are highly fractal and tape-like structures are also present.

### Catalytic Performance of Optimized AuNS

**Figure 8:**
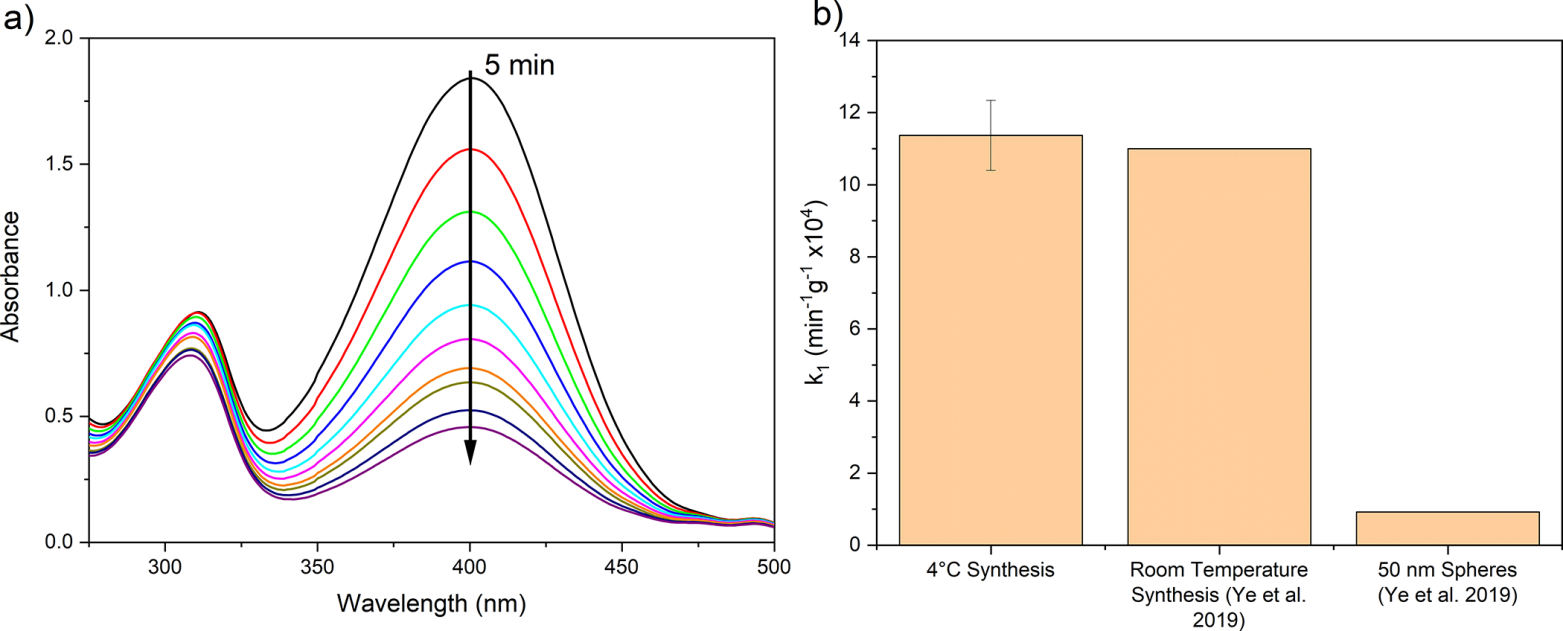
Catalytic activity of optimized AuNS. (a) UV–vis spectra showing the reduction of 4-nitrophenol by sodium borohydride in the presence of AuNS over 5 min. (b) A comparison of the mass normalized rate constant, *k*_1_, between AuNS produced with the optimized synthetic protocol from this work (*n* = 3), and AuNS and AuNP published by Ye et al.^8^

The ultrathin nature of AuNS affords an exceptionally large surface area-to-volume ratio. Furthermore, the high proportion of edges leads to a high edge-to-surface ratio that is known to impart high catalytic activity. Our previous studies into this synthetic protocol showed that the AuNS were just 2 atoms thick, suggesting that every atom is presented at the particle surface and available for reaction.^8^ Herein, we assessed the catalytic performance of the AuNS synthesized via the newly optimized synthetic procedure presented above, which incorporates significantly enhanced mass yield and reproducibility (*T* = 4 °C, *t*_f_ = 1 s, *t*_sc_ = 30 s, *t*_synth_ = overnight, 1000 *g* clean), and compared it to our previously reported work.^8^ The reduction of 4-nitrophenol to 4-aminophenol by sodium borohydride in the presence of AuNP served as a model reaction. The reaction progression was marked by a color change from yellow to transparent and was monitored by the absorbance at 400 nm (Figure 8a). Pseudo first-order kinetics were observed, and the rate constant *k*_app_ was found from the gradient of ln(*C_t_*/*C*_0_) over time. The mass normalized rate constant *k*_1_ was found to be (11.4 ± 1) × 10^4^ min^–1^ g^–1^, in good agreement with the previously reported value of 11 × 10^4^ min^–1^ g^–1^ and more than 10-fold higher than that of 50 nm AuNP, which have *k*_1_ ∼ 1 × 10^4^ min^–1^ g^–1^ (Figure 8b).^8^ Furthermore, our value for the mass normalized rate constant offers a nearly threefold improvement over the 15 nm-thick gold AuNS prepared by Zhang et al.^33^

## Conclusions

We have explored the mechanism of ultrathin AuNS formation using MO as a confining agent. The time-resolved spectral analysis of the reaction between HAuCl_4_ and MO showed that the MO is degraded over ∼30 s and obeys pseudo first-order reaction kinetics. FTIR and NMR studies of the interaction showed that the MO is broken down to produce protonated dimethylamine and 4-DBSA (compounds **4a** and **4c**, Scheme 1). When AuNS synthesis was attempted using 4-DBSA rather than MO, although the formation of spherical AuNP was suppressed, AuNS yield was low and with low structural purity. This implies that the 2D AuNS formation is a result of a combination of the ability of the MO to self-assemble at very low concentrations and its chemical oxidation in situ. This indicates a more complex interplay between HAuCl_4_ and MO and its reaction products, ultimately leading to the formation of 2D AuNS.

Optimization of the AuNS synthesis conducted at room temperature showed that a delay of 30 s between the addition of HAuCl_4_ and SC to MO, coupled with “slow” injections of solutions, gave improved AuNS yield. Time-resolved UV–vis analysis of the synthesis showed the simultaneous formation of the 2D and 3D materials, with the collected product determined by the centrifugation conditions. To obtain AuNS structures, relatively gentle centrifugation conditions should be used, typically ∼1000 *g*. Time-resolved UV–vis monitoring of sheet synthesis showed the synthesis to be complete after ∼8 h, with AuNS structures observable after 1 h. The yield of AuNS obtained at room temperature was dramatically improved by lowering the synthesis temperature to 4 °C. This provided a threefold increase in AuNS production over the slow addition room temperature system and a near 16-fold increase in AuNS production over the fast addition room temperature synthesis. Our optimized synthesis is reproducible and led to a product with enhanced catalytic activity compared to AuNP. These 2D AuNS are of interest for catalytic, enzymatic, and diagnostic applications, where the large surface area-to-bulk ratio offers a significant enhancement in activity per unit mass of gold.

## Supporting Information Available

Additional UV–vis spectra and images of MO + HAuCl_4_ reaction, NMR spectra of MO + HAuCl_4_ reaction product, FTIR spectra of 4-DBSA, substitution of MO for 4-DBSA in AuNS synthesis, investigation of influence of centrifugation speed on collected product, and investigation of the influence of *t*_synth_ on the collected product (PDF). Data used in the figures of this paper are openly available from the University of Leeds data repository https://doi.org/10.5518/1151.

## Abbreviations

AuNS: gold nanosheet
AuNP: gold nanoparticle
MO: methyl orange
SC: sodium citrate
TEM: transmission electron microscopy
4-DBSA: 4-diazobenzenesulfonic acid
SPR: surface plasmon resonance
OD: optical density
UV–vis: ultraviolet–visible spectroscopy
FTIR: Fourier-transform infrared spectroscopy
^1^H-NMR: proton nuclear magnetic resonance
