# Supplementary material for: Spectrophotometric Analysis and Optimization of 2D Gold Nanosheet Formation
Source: J Phys Chem C Nanomater Interfaces. 2023 Feb 6;127(6):3067–76. doi: 10.1021/acs.jpcc.2c07582 (PMC9940192; doi:10.1021/acs.jpcc.2c07582)
Supplement: Supplementary file 1 — jp2c07582_si_001.pdf [file jp2c07582_si_001.pdf]

Supporting Information - Spectrophotometric Analysis and Optimization of 2D Gold Nanosheet Formation

Joseph Fox[1], George Newham[1], Richard J. Bushby[1,2], Elizabeth M.A. Valleley[3], P. Louise Coletta[3] and Stephen D. Evans[1].*

[1]Molecular and Nanoscale Physics Group, School of Physics and Astronomy, University of Leeds, LS2 9JT, United Kingdom

[2]School of Chemistry, University of Leeds, Leeds, LS2 9JT, United Kingdom

[3]Leeds Institute of Medical Research, Wellcome Trust Brenner Building, St James's University Hospital, Leeds, LS9 7TF, United Kingdom

*Corresponding author E-mail: s.d.evans@leeds.ac.uk

1 Supporting Information

1.1 Additional MO/$HAuCl_4$ Interaction Analysis

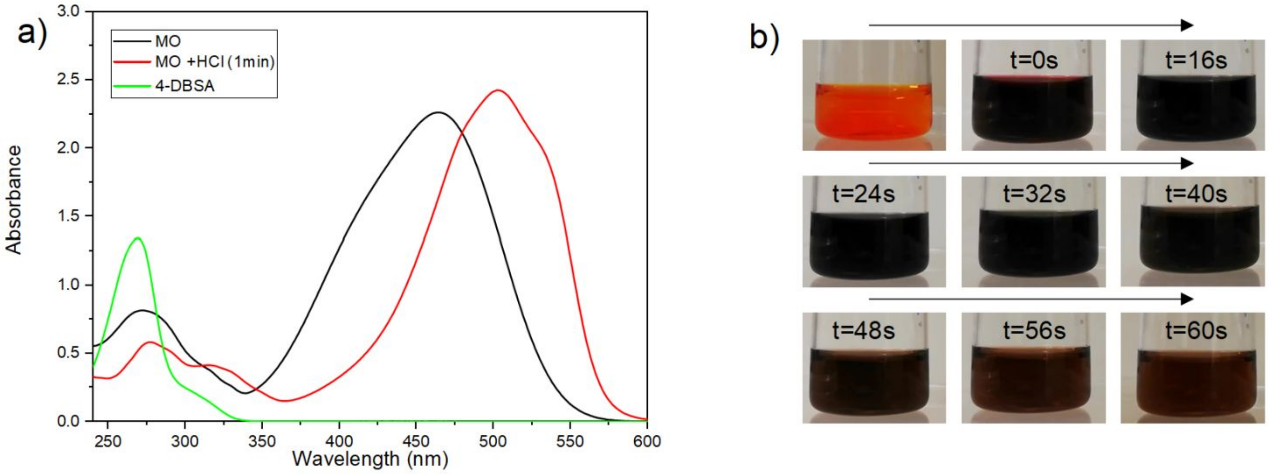

Figure S1 Additional MO/$HAuCl_4$ Interaction Analysis. a) UV-Vis spectra of MO, MO 1 minute after addition of HCl and spectra of 4-DBSA. b) Photographs of colour change observed before and after $HAuCl_4$ added to MO solution over a 1-minute period.

1.2 ^{1}H-NMR spectra

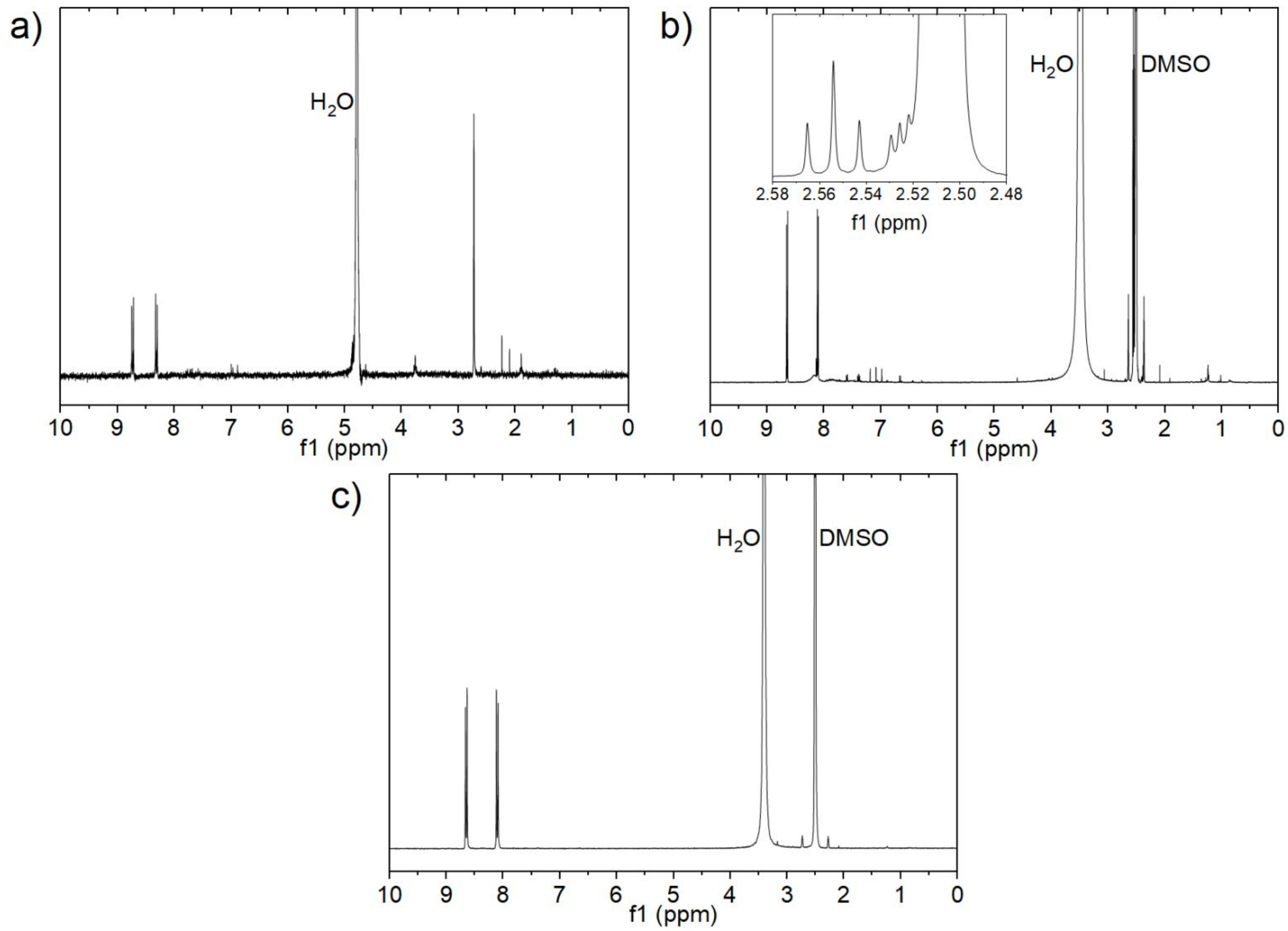

Figure S2 1H-NMR Spectra. a) ^{1}H-NMR spectrum of the product of the reaction between MO and $HAuCl_4$ in D_2O (obtained under equivalent conditions to those used in the AuNS preparation). b) ^{1}H-NMR spectrum of the product of the reaction between MO and $HAuCl_4$ in d_6-DMSO (obtained under equivalent conditions to those used in the AuNS preparation). Inset: expansion showing the methyl triplet of the dimethylammonium resolved from the solvent peak. c) ^{1}H-NMR spectrum of authentic 4-diazobenzenesulfonic acid (prepared by diazotization of sulphonamide) in d_6-DMSO.

1.3 FTIR Spectra of 4-Diazobenzensulfonic Acid

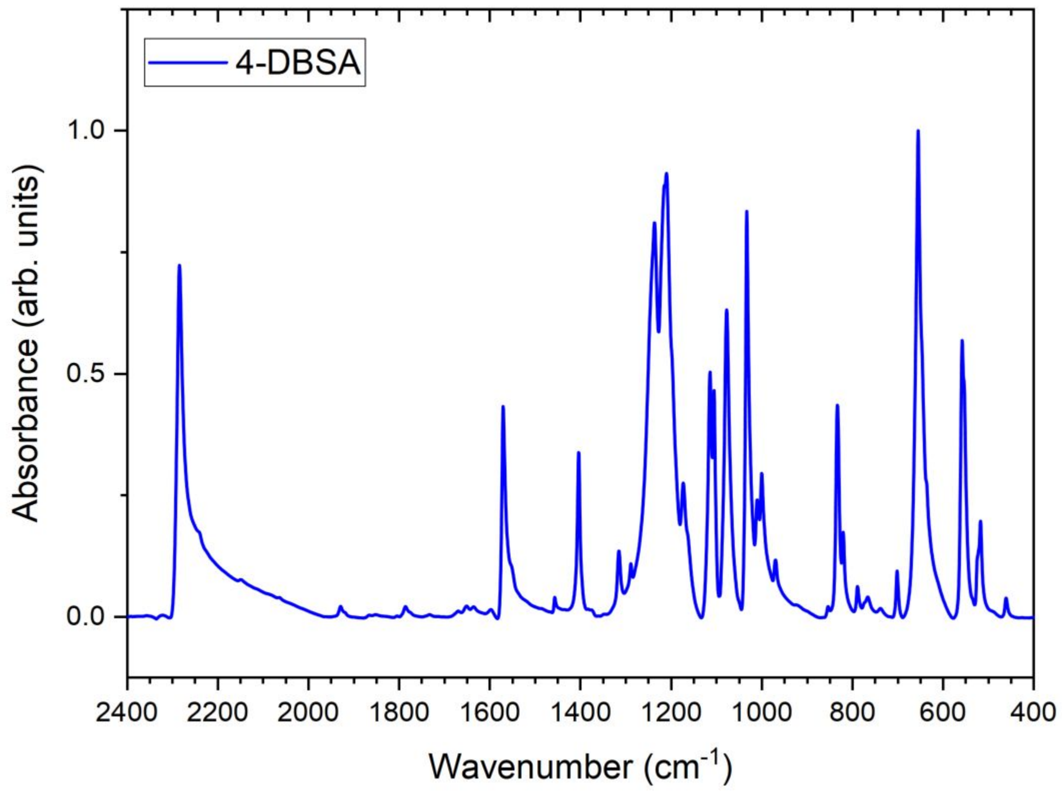

Figure S3 FTIR of 4-DBSA.

1.4 Use of 4-Diazobenzenesulfonic Acid as Shape Directing Agent

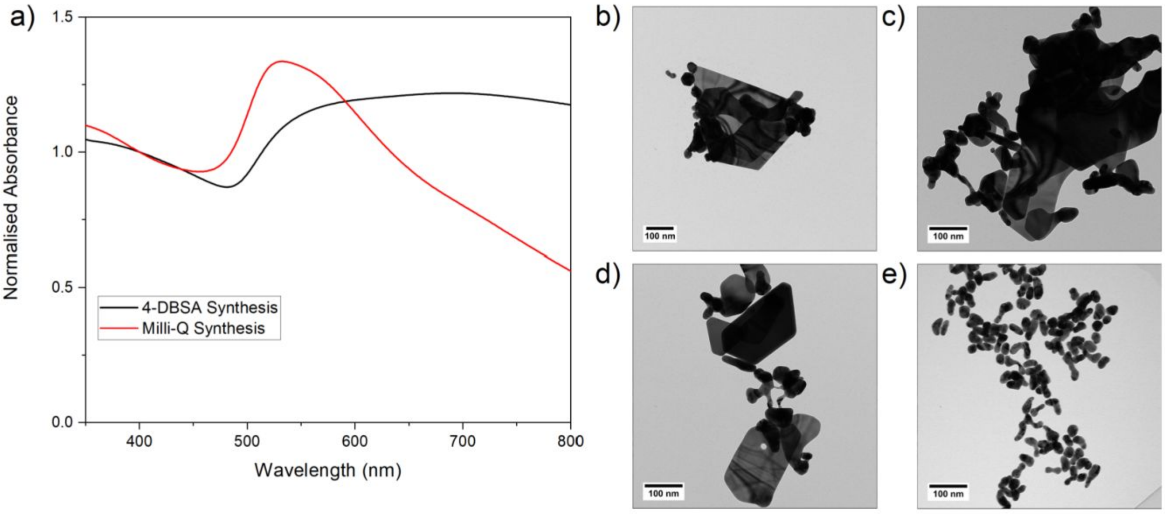

Figure S4 Substitution of MO for 4-DBSA or Milli-Q in synthesis (t_{sc} = 30 s, t_f = 3 s, t_{synth}=17 hrs, centrifugation 3x at 1000g for 10 minutes). a) Post-clean UV-Vis spectra of samples synthesised using 4-DBSA or MQ in the place of MO, normalised to OD at 400nm. T12 TEM images of post-clean samples which were synthesised using b)-d) 4-DBSA or e) Milli-Q water. For 4-DBSA the UV-Vis spectrum was comparable to that for MO (**Figure S4**a) with a trough in absorbance at ~ 500 nm and subsequent increase at higher wavelengths which suggest the formation of AuNS. However, the absorbance at 800 nm and ratio of OD_{800}:OD_{400} is much lower than that observed when MO was used as the shape directing agent (1.16 for 4-DBSA and 1.33 for MO). The characteristic wrinkling of the sheet surface (a hallmark of ultrathin 2D material) was observed in TEM images, although a significant quantity of 3D material was also present and no standalone AuNS were obtained (**Figure S4**b– d). When MO was substituted for Milli-Q water (i.e. no shape directing agent), we observe a spectra with SPR peak at 530 nm and low absorbance in the NIR region, all of which are key characteristics of 3D gold nanospheres. TEM imaging of the control synthesis in **Figure S4**e shows no 2D material. Instead, we obtain quasi-spherical gold nanoparticles which agree with the spectral data for this synthesis. Notably, without normalisation of UV-Vis, the 4-DBSA synthesis yielded a 10-fold increase in gold mass compared to the Milli-Q control, with OD_{400} = 1.52 and OD_{400} = 0.14 for the 4-DBSA and Milli-Q respectively. Hence, we have shown that the gradual, in-situ formation of 4-DBSA via the MO and $HAuCl_4$ reaction prior to SC addition is critical in the formation of standalone ultrathin gold sheets, rather than the direct replacement of MO with 4-DBSA in the synthesis.

1.5 Collection of 2D AuNS

Figure S5a shows the spectra obtained from t_{sc} = 0 s product, after cleaning at different centrifugation speeds. The spectra have been normalised to the absorbance at 400 nm to allow visualisation of the changes in spectral shape to be made clear. We observe that higher spin speeds i) lead to higher yield of total gold product (OD_{400}) collected and ii) lead to a slight reduction in absorbance in the longer wavelength region (associated with 2D material). Consistent with the relatively small changes in the UV-Vis spectra, TEM imaging revealed that for t_{sc} = 0 s the higher centrifugation speeds did not yield a significant change in particle morphology. Both 1000 and 4600 g yielded a mixture of 2D sheet like material with 3D aggregates (**Figure S5**a inset shows a 1000 g example).

Figure S5b shows the equivalent t_{sc} = 30 s product spectra. It demonstrates that stronger spin speeds generate spectra more akin to 3D AuNP, characterised by lower NIR absorbance and the emergence of a SPR peak ~535 nm. As before, the OD_{400} increases with spin speed. TEM images from the t_{sc} = 30 s regime when cleaned at 1000 (**Figure S5**c), 3000 (**Figure S5**d) and 4600 g (**Figure S5**e) show that, unlike the t_{sc} = 0 s regime, the morphology subtly changes. Corresponding TEM shows high centrifugation speeds gives a tape-like material. In some cases, these tape-like particles display a dense 3D head, which agrees with the presence of the SPR band in the particle spectra. At lower spin speeds, particle spectra showed improved NIR absorbance and yielded larger structures with 2D regions. For t_{sc} = 0, the ratio of OD_{800}:OD_{400} decreased from 1.27 to 1.13 when the centrifugation speed was increased from 1000 to 4600 g, while the ratio decreased from 1.05 to 0.84 for t_{sc} = 30 s indicating a reduction in the relative proportion of 2D AuNS.

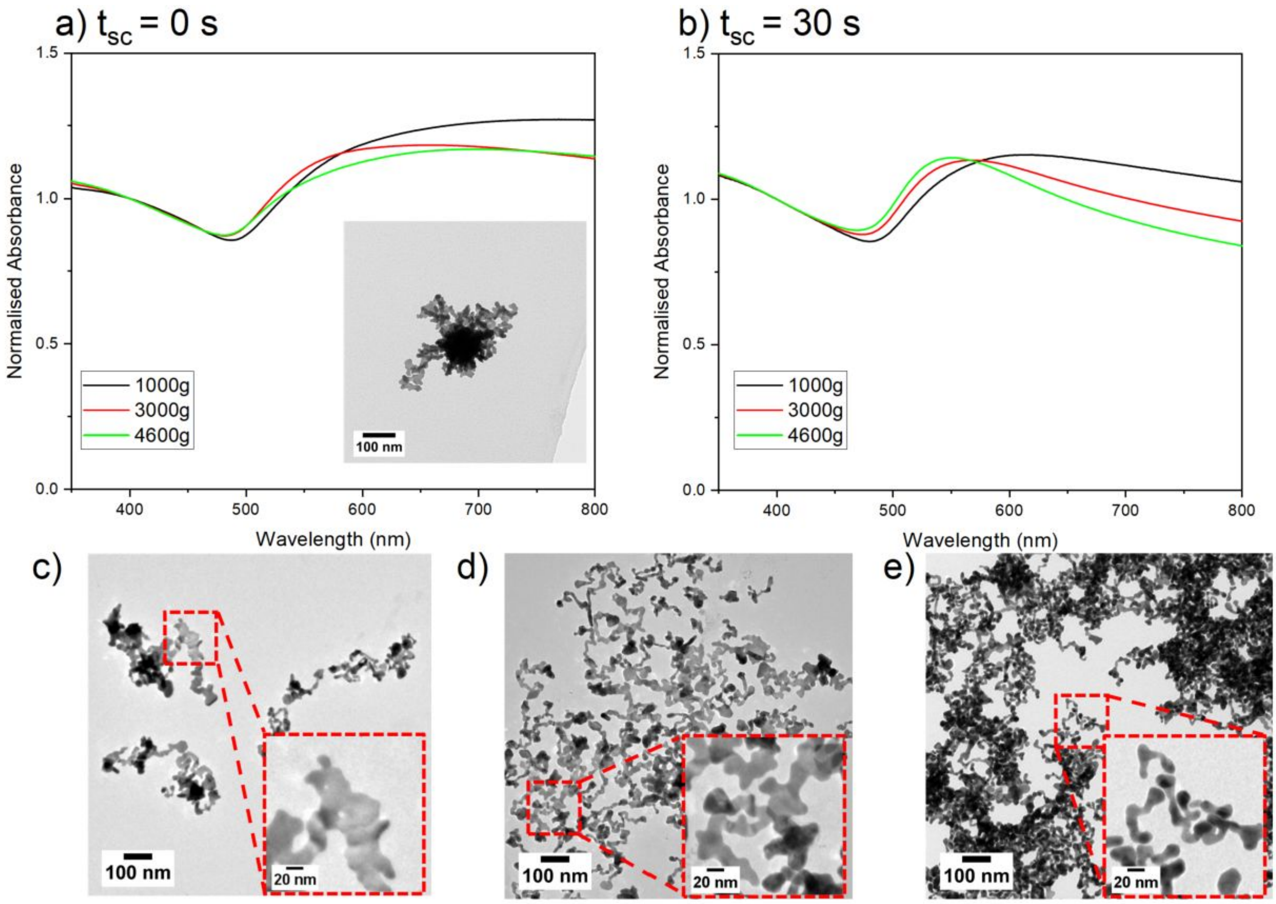

Figure S5 Influence of centrifugation speed on collected product. a) Post-clean UV-Vis absorbance spectra of samples synthesised using t_{sc} = 0 s, t_f = 1 s and t_{synth} = 16 hr 30 mins after 3, 10 minute centrifugation cycles at different speeds. Spectra normalised at 400 nm to highlight changes in spectral shape, OD_{400} were 0.57, 1.52 and 1.81 for the 1000g, 3000g, and 4600g spin conditions respectively. Inset shows post clean TEM image from 1000g cleaned sample. b) Post-clean UV-Vis absorbance spectra of samples synthesised using t_{sc} = 30 s, t_f =1 s and t_{synth} = 15hr 46 mins after 3, 10 minute centrifugation cycles at different speeds. Spectra normalised at 400 nm to highlight changes in spectral shape, OD_{400} were 0.11, 0.93 and 1.50 for the 1000g, 3000g, and 4600g spin conditions respectively. c) d) and e) show F20 TEM images of samples which received 1000, 3000 and 4600g spins respectively, in the t_{sc} = 30 s regime.

1.6 Effect of Total Synthesis Time (t_{synth}) on AuNS Formation

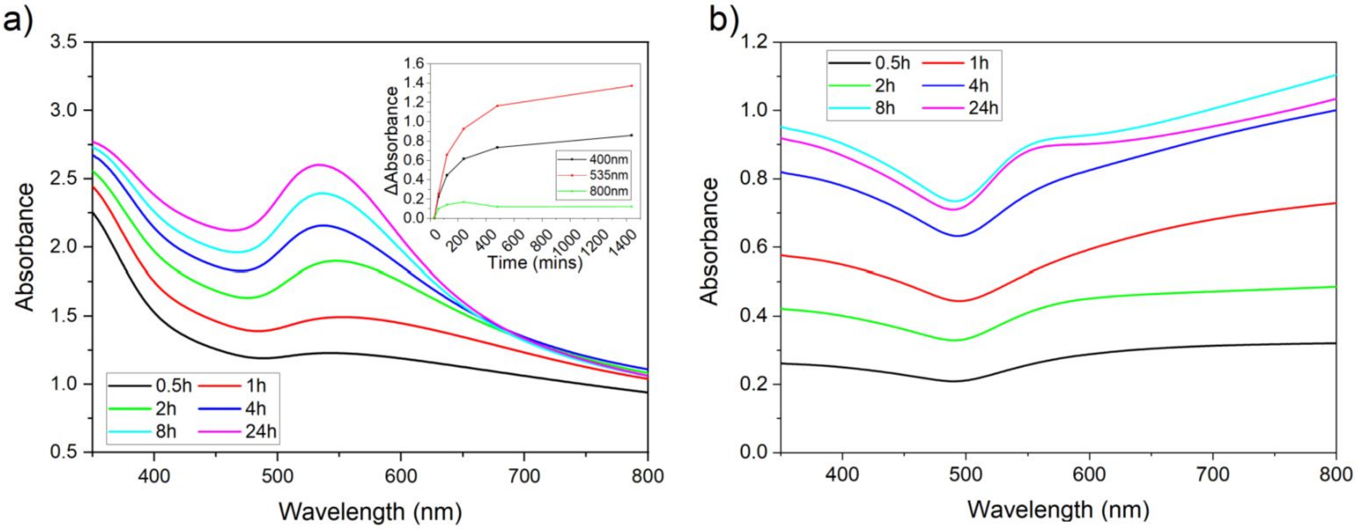

Figure S6 Influence of t_{synth} (t_{sc} = 30 s, t_f = 3 s, centrifugation 3x at 1000g for 10 minutes). a) Pre-clean UV-Vis absorbance spectra with inset showing change in absorbance of mother solution at 400, 535 and 800 nm with respect to the first time point, plotted against time. b) Post-clean UV-Vis absorbance spectra of synthesis stopped at a range of time points.
